# Cystoscopy as Adjuvant in Surgery, with an Atlas of Cystoscopic Views and Concomitant Text for Physicians and Students

**Published:** 1910-12

**Authors:** 


**Cystoscopy as Adjuvant in Surgery, with an Atlas of Cystoscopic Views and Concomitant Text for Physicians and Students.** By Staff Surgeon **Dr. O. Rumpel,** Lecturer in Surgery at the University of Berlin. Authorized English Translation by **P. W. Shedd,** M.D., New York. Octavo, pp. 131. With illustrations in color on 36 plates and 22 textual figures. New York: Rebman Company. (Half leather, $8.50.)

The announcement that Rebman was to issue an atlas of cystoscopic views carried with it the sense of satisfaction that, so far as the mechanic production was concerned, there would be little to criticise and that the book would be of value to the profession.

This firm's productions are not thrown off helter-skelter irrespective of merit or need, but are issued because there is a definite reason for their existence. Cystoscopy as an adjuvant in surgery, is no exception to this rule, and it is surprising that so much material of inestimable value has been placed within the reach of the general medical public in so compact and such artistic form. There is lacking that glitter of generalities which is so tempting of insertion in an atlas on any subject and so particularly irresistible of impulse in a work on cystoscopy because of the mystery, the wonder, the art of the subject. The book is by Staff Surgeon Dr. O. Rumpel, lecturer in surgery at the University of Berlin, the present authorised English translation being by Dr. P. W. Shedd, of New York, who has done a most excellent bit of work and ably carried out the ideas of the author. As for the rest, the actual production, the giving of this excellent work to the world, the credit belongs entirely to Rebman. A less careful publication would have made the book absolutely useless as an aid in diagnosis and a guide to recognition of pathologic conditions within the urinary bladder. With a maximum of commercialism and a minimum of art its only excuse for existence would be its extreme of caricature. Fortunately, its American publishers are the Rebman Company, and those of us who are familiar with their standards are content to accept it as the best there is.

A careful review of the book—and it is a book rather than an atlas, since we are ingrown of the belief that an atlas is a large bulky and cumbersome tome of many pictures and little words,—reveals its scientific values. In his preface the author speaks of the illustrations of cystoscopic views, which, by the way, are the work of Max Queisser, and gives grateful recognition for "their beauty of execution" to his publishers.

The artistic excellence of the colored plates is exquisite; their fidelity to existing conditions is merely amazing. Simply to give careful study of these plates before embarking on one of those wonderful voyages of discovery which the cystoscopist takes, would be of incomparable value to one not technically perfect in the use of the cystoscope, and he would be enabled to get a satisfying and comprehensive grasp of a pathologic condition within the bladder which otherwise would be to him a weird and distorted series of grotesqueries; of strangely grouped hills and valleys; of pink landscapes with tiny, wavy, blood-red rivers; of glares and shadows; of queer combinations of pinks and reds and yellows; of tears of blood from a rift in the red and swollen landscape which is passing before his wonder-widened eye. All these would have significance of scientific value after a memorising view of the plates in Rumpel's book. He would recognise in the picture of a blood-red sky behind the jagged crags of precipitous mountain tops and the low lying glaciers, the presence of a highly inflammatory bladder caused by a large, rough calculus; the graceful undulations of filmy, mossy projections into the field of vision would be recognised for what they are,—papillomatous growths.

One might, without apology, go on to the uttermost margin of literary limits in the description of the marvelous beauties of these colored plates and still be within the bounds of extravagance. They are artistically superb; scientifically they are wondrously exact, and there has been maintained that fine degree of perspective which gives an anatomic reproduction its exact value. In this respect the plates are far superior to those in Nitze's "Lehr buch der Kystoskopie," which, while colorful and helpful, maintains that flatness of surface so beloved of the Japanese artist, while the plates in Georges Luys's "Exploration de l'Appariel Urinaire" lack the vivid coloring which greets the eye of the operator. These comparisons are not made with any idea of minimising the importance or value of the works by Nitze and Luys, but to accentuate the superiority of the English book under consideration as a work of art and accuracy. The text in the Rumpel book is not extensive; what there is of it is expression of the personal experience of the author and is, therefore, of exceptional interest and follows closely the conditions illuminated in the plates. The only discrepancy noted is the absence of syphilitic lesion of the bladder. The text treats of congenital anomalies, calculi, cystitis, foreign bodies, hypertrophy of the prostate, the prostate tumors, and testing renal function.

"Rumpel's Cystoscopy" is one of the most useful and valuable books which the Rebman Company has published.

